# Perception of Learning Environment Among Anaesthesiology Residents During the Pandemic in a Tertiary Hospital in India: Comparative Cross-Sectional Study

**DOI:** 10.5152/TJAR.2022.21373

**Published:** 2022-04-01

**Authors:** Sree E. J. Kumar, Gautham Ganesan, Gautham Anbu, Thirumalai Priya, Venkatesh Selvaraj

**Affiliations:** 1 Department of Anaesthesiology, Sri Ramachandra Institute of Higher Education and Research, Chennai, India

**Keywords:** Anaesthesiology, educational measurement, learning, questionnaire, residency

## Abstract

**Objective:**

The coronavirus disease pandemic has affected the postgraduate educational system infusing online teaching resulting in a blended teaching-learning experience especially in the field of anaesthesiology. Hence, we conducted this study to evaluate the effect of the introduction of blended learning methods on students’ perception of the learning environment among different years of anaesthesia residency training.

**Methods:**

We invited 44 residents belonging to 3 years (Y1, Y2, and Y3) of anaesthesia residency to complete the Dundee Ready Education Environment Measure questionnaire. This study was done during the coronavirus disease pandemic after 6 months of incorporation of blended learning methods into the teaching program. The first-year cohort was not exposed to traditional anaesthesia teaching. The student's perception of learning was assessed after 6 months of change in the teaching method. The total Dundee Ready Education Environment Measure scores and the individual domains were compared among the 3 years.

**Results:**

There was a significant difference in the corrected Dundee Ready Education Environment Measure score between Y1 (154.2 ± 20.73 [145.11-163.29]) and Y2 (138.27 ± 22.12 [125.2-151.34]) with *P*  = .027. There was no significant difference in the individual domains.

**Conclusion:**

Higher score in the first-year residents suggests that it is appropriate to introduce blended learning from the beginning of the course rather than slowly merging with the existing traditional face-to-face teaching-learning methods.

**Main Points**

- Dundee Ready Education Environment Measure questionnaire is valid to be used among anaesthesiology residents.
- Perceptions of the learning environment are reported during the pandemic and it varies among the different years of residency.
- Dundee Ready Education Environment Measure correlates with anaesthesiology faculty assessment score.
- Faculty assessment correlates with the residents’ perception of learning and atmosphere.

## Introduction

With the onset of coronavirus disease (COVID) pandemic, medical education has changed dramatically, with the distinctive rise of e-learning, whereby teaching is undertaken remotely and on digital platforms. The essentials of medical education, especially training anaesthesia residents, need to turn to a blended model of education, which has been referred to as phygital mode. Blended teaching provides the opportunity to have face-to-face teaching, which is an essential of clinical bedside teaching, and the incorporation of online teaching aids to improve teaching and learning.^1^ Previous literature has shown promising improvement in postgraduate learning experience with blended teaching methods^2,3^ compared to the traditional medical education system. The teaching methods affect the learning experience and perception of the students to a greater deal.^4^ The Dundee Ready Education Environment Measure (DREEM) was a questionnaire-based tool to evaluate educational environments of medical schools and other health training settings, and it was considered as the most suitable such instrument.^5^ It is made of 50 statements, grouped into 5 domains, each with a 5-point scale ranging from strongly disagree to strongly agree. Supervision during residency forms a major factor for training and can also affect the learning environment (LE). de Oliviera Filho et al’s^6^ instrument for measuring faculty anaesthesiologists’ supervision of anaesthesiology residents (faculty assessment score [FAS]) is one such validated instrument for anaesthesiology.^6^

Coronavirus disease pandemic has given an option to conduct this study that involves 3 cohorts of postgraduate trainees belonging to 3 different years of residential training, namely the first cohort that includes residents who are exposed to blended teaching methods without prior exposure to traditional teaching methods, the second cohort includes second-year trainees who had 1 year of previous traditional teaching methods and then exposed to the blended teaching methods, and the third cohort includes the third-year trainees who had 2 years of previous traditional teaching methods and then exposed to the blended teaching methods. This study was designed as a prospective observational cohort study to compare the perception of learning experience in terms of DREEM score within the year of residency training among anaesthesia residents after the introduction of blended learning into the curriculum. The secondary aim is to compare individual component domain of DREEM score among the years of residency and faculty assessment score among the 3 years of residency and to check for correlation of FAS and DREEM.

## Methods

After obtaining Institutional ethics committee approval, we invited 44 anaesthesiology residents belonging to 3 years of anaesthesia residency to complete the DREEM questionnaire, FAS, and a survey of baseline demographic, educational, and economic characteristics. This study was done during April 2021, during the COVID-19 pandemic after 6 months of incorporation of blended learning methods into the residential teaching program. The study population includes 3 cohorts of anaesthesia residents which are as follows:

Cohort 1 (Y1) includes residents who are exposed to blended teaching methods without prior exposure to traditional anaesthesia teaching methods.

Cohort 2 (Y2) includes second-year trainees who had 1 year of previous traditional teaching methods in anaesthesia and were then exposed to the blended teaching methods.

Cohort 3 (Y3) includes the third-year trainees who had 2 years of previous traditional teaching methods in anaesthesia and were then exposed to the blended teaching methods.

Written consent from the participants was obtained. The questionnaire was sent to the participants by the primary author in the form of an online form through email and was given 24 hours to reply. The submitted form was devoid of resident identification. The DREEM questionnaire has 5 domains—students’ perception of learning (SPL), students’ perceptions of teachers (SPT), students’ academic self-perceptions (SAP), students’ perceptions of atmosphere (SPA), and students’ social self-perceptions (SSP). According to Roff et al.,^7^ individual items with a mean score of 3 or greater reflect a positive educational environment and are considered areas of strength for a school; values between 2 and 3 reflect areas that are neither strengths nor weaknesses but identify areas that could be enhanced; items with a mean score below 2 are considered areas of weaknesses for a school of medicine.^7^

The DREEM yields a global score of up to 200 with its 50 items combined and has the following 5 subscales.

1. Students’ perception of learning that addresses students’ views of aspects of the teaching activities, such as whether they receive clear course objectives and whether learning is student-focused and encourages active learning rather than being teacher-centered and stresses factual learning;
2. Students’ perceptions of teachers that address students’ views of the qualities of teachers, including their communication skills, whether they provide feedback to students and patients, their level of knowledge, and their level of preparation for classes;
3. Students’ academic self-perceptions that include students’ views of the learning strategies and problem-solving skills they have developed to prepare themselves for their profession;
4. Students’ perceptions of atmosphere that includes items addressing how relaxed the atmosphere is during lectures and ward teaching, whether teaching activities are motivating for students and whether there are opportunities for students to develop interpersonal skills;
5. Students’ social self-perceptions that address students’ views of the support systems available to those who become stressed, the school’s accommodations for students, the quality of campus social life, and whether students can find friends at school.

A corrected total DREEM score and corrected SAP were derived as the first-year residents cannot answer the question “Last year’s work has been good preparation for this year’s work.” The FAS included 9 items for evaluating the quality of faculty supervision of anaesthesiology residents. The instrument utilizes a 4-point Likert-type scale (1 = never to 4 = always).

### Power Analysis

As mentioned in the previous publication,^8^ the average DREEM scores are between 105 and 170. As the total number of residents is 44, we expected to have 90% power at the .05 significance level to detect a change of 10 points.

### Statistical Analysis

We compared residents on baseline characteristics across years of residency using χ^2^ tests. The DREEM questionnaire overall score and average individual domain scores were calculated (i.e., score divided by the number of questions) to enable comparison among the domains. The overall standardized score and each domain score ranged from 0 to 4. Nonparametric test was used in view of the small sample size. *P* value of <.05 was considered significant. All analyses were completed using PSPP (Version 3, 29 June 2007, Free Software Foundation, Inc., Boston, Mass, USA). Cronbach’s α was done to check the inter-relatedness of the items in each domain. Kendall’s *W* value was used to measure agreement among the students. 

## Results

**Figure 1.**
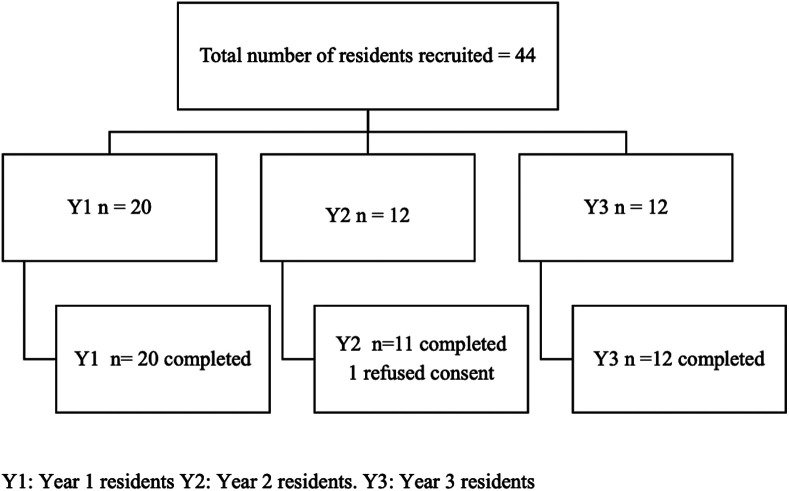
Study flow diagram.

Of the 44 residents invited to complete the DREEM questionnaire, 43 completed, including 20 first-year (Y1) residents, 11 second-year (Y2) residents, and 12 third-year (Y3) residents, and 1 resident refused consent as shown in Figure 1. 

Baseline resident characteristics were compared across year of residency, and age was found to be significantly different among the groups (Table 1). There was no significant difference among the year of residency with regard to gender, relationship status (*P*  = .278), national eligibility test rank (*P*  = .230), availing student loan, stress due to loan, number of hours of work, and number of cases done (Table 1). 

All DREEM questionnaire domains had a Cronbach’s α > 0.7 (Table 2), suggesting that questions within each domain are correlated and that internal consistency is acceptable. Overall DREEM scores within each domain by year of residency are shown in Table 3. There was a significant difference in the SPT and SPA between the 3 cohorts in terms of the individual domain comparison. There was a significant difference between the years of residency in the corrected DREEM score (*P*  = .042).

**Figure 2.**
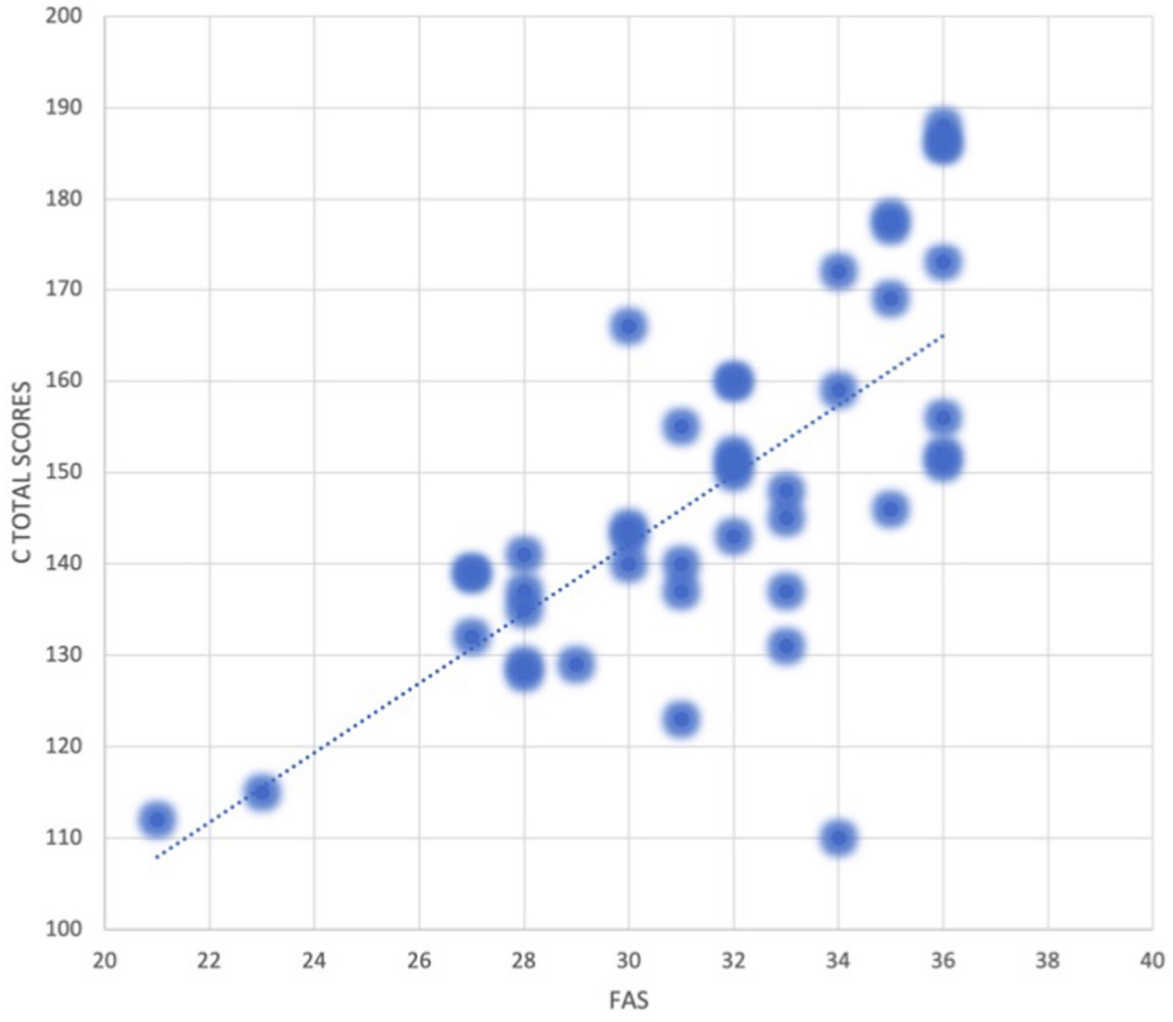
Correlation between faculty assessment score (FAS) and corrected total (C.Total)

On comparison of individual domains among the 2 groups, there was a significant difference between Y1 and Y2 in corrected DREEM score (*P * = .027) and SPL as shown in Table 4. The average scores by the domains and total scores by the year of residency are given in Table 5. The FAS correlated with corrected total (*R* ^2^ = 0.49, *P* <.001) (Figure 2), SPA (R^2^ = 0.43, *P* < .001), and SPL (R^2^ = 0.41, *P* < .001). The *R* ^2^ values were lower for SPT (0.30, *P* < .001), SAP (0.15, *P*  = .011), and SSP (0.12, *P*  = .022). 

To summarize, there was a significant difference in SPT and SPA among the 3 cohorts, with cohort 1 (Y1) having a higher score significantly than the other 2 cohorts. The total corrected score is statistically significantly different among the 3 cohorts. In inter-group comparison, the students’ perceptions of learning are statistically significantly different between cohorts 1 and 2 as shown in Table 4. Table 5 shows the average value of each question and shows a significant difference among the domains with strong agreement among students.

## Discussion

With the emergence of the pandemic, there is a rapid change in the LE,^9^ with an increase in online learning components resulting in blended teaching method along with the face-to-face traditional teaching method. To our knowledge, this is the first study to analyze the anaesthesiology resident’s perception of LE during the pandemic period. We used the DREEM questionnaire that was shown to be an internally reliable instrument for measuring students’ perception of the educational climate.^7^

With multiple factors affecting the LE and smaller number of residents, analyses rely on the use of questionnaires and their interpretation. Colbert-Getz et al^10^ reported that no gold standard exists for assessing the residents’ perceptions of the LE. They found that the LE tool by Accreditation Council for Graduate Medical Education (ACGME) was comparatively more valid among LE tools. The other questionnaires used are the Postgraduate Hospital Educational Environment Measure (PHEEM),^11^ Dutch Residency Educational Climate Test (D-RECT), and DREEM. But, the majority of the medical educational environment instruments do not have a theoretical framework.^12^ Analyses of LE tools have shown that though D-RECT was much better supported, DREEM has been more commonly employed.^10^ Individual postings of residents with collaboration only in the classroom, non-applicability of certain parts of the questionnaire made it difficult to use ACGME, PHEEM, and D-RECT in our institute. Dundee Ready Education Environment Measure has been previously used to study the perception of medical students in the Indian context.^13^ Also, the DREEM questionnaire could be mapped to the Moos theoretical framework, where each LE environment—irrespective of the type of setting—can be described by personal development or goal direction, relationship dimensions, and system change dimensions. The internal consistency of DREEM was checked in our study for 2 reasons: (1) a correction to the DREEM score was done in our study in the DREEM score, as the question of how the present year was compared to the previous year was not applicable to the first-year residents; (2) the internal consistency of the scales has been variable, and studies have varied in their conclusion.^5,14,15^ The internal consistency of DREEM was checked in our study. Cronbach’s alpha provides a measure of the internal consistency of a test and is expressed as a number between 0 and 1, with acceptable values between 0.7 and 0.90. The measurement of Cronbach’s alpha adds validity to the interpretation of data.^16^ A correction to the DREEM score was done in our study in the DREEM score, as the question of how the present year was compared to the previous year was not applicable to the first-year residents. The first-year students have been exposed to the same traditional face-to-face teaching method in the undergraduate course, though the postgraduate teaching expects more in-depth and subject-oriented focused knowledge sharing. Blended learning methods should be introduced into the earlier period of the course rather than merging with the existing traditional method.^17^

Previous study showed that there was no difference in terms of perception of LE among the 3 years of anaesthesia residency when they are subjected to the same teaching-learning methods throughout their tenure of residency.^18^ But, in our study, the impact of introduction of blended teaching methods into the curriculum among the 3 years of anaesthesia resident program was studied in terms of students’ perceptions of learning. Changes in teaching training module have shown to create a change in the students’ perceptions of LE.^16^ In our study, the 2 cohorts had a reasonable number of years of traditional anaesthesia residential training compared to the first cohort in which the residents are subjected only to the blended teaching method. 

Our study has shown that the students’ perceptions of LE were better with residents exposed to blended teaching methods compared to the other year of residents who are exposed to both traditional and blended teaching methods. The first-year students’ perceptions of teacher were positive than the students of other 2 years, though not significant. The postgraduate training gives more student–teacher interaction compared to undergraduate training which can be due to low student-to-teacher ratio, more clinical time with the teachers, specialized area for in-depth knowledge acquisition. Hence, the author felt that the first-year students had higher score for the student’s perception of teacher domain.

The next domain that has shown more positive score was perception of learning atmosphere among the first-year students. By using a combination of digital instruction and one-on-one face time in blended learning methods, students can work on their own with new concepts which free teachers up to circulate and support individual students who may need individualized attention. Rather than playing to the lowest common denominator—as they would in a traditional classroom—teachers can now streamline their instruction to help all students reach their full potential,^19,20^ thus providing a favorable atmosphere to the students. In addition to the findings, blended teaching methods have also shown to improve the outcomes and achievements of teachers.^21^

The current generation of anaesthesia residents are primarily “Millennials.”^22^ Millennial learners have been described as having shorter attention spans, crave interactivity, and struggle with reflective endeavors. Students’ social self-perceptions section of the DREEM has shown correlation with subjective happiness.^23^ The least score in our study by Y2 and Y3 was in SSP, like previous studies.^8,24,25^ This can be explained by the fact that in anaesthesiology training, in the operating room, there is lesser time for interaction with peers. But a lower Kendall’s *W* value shows that the agreement among the students for the rating was less.

The mean scores obtained in our study can be classified as more positive than negative.^26^ The overall score in our population was higher than that observed by Riveros-Perez et al^8^ and de Oliveira Filho et al^15^ But this could be due to differences produced by the pandemic and variation in sociocultural factors. The scores given by the first-year residents can also be taken as the expectation of the resident as they are yet to completely understand the LE. They might not recognize the bi-directional nature of the relationship between student environment and LE.^27^ Our study shows that the expected LE was not significantly different from that of the final year.

The limitation of the study is its observational nature. It is not known whether the 6 months of blended learning is sufficient to induce changes in perception of the students. The complexity of the LE makes it important to do repeated measurements.

To conclude, the authors felt that there was more positive scoring in the naive first-year residents after introduction of blended learning methods, and hence, it will be appropriate to introduce blended learning from the beginning of the course rather than slowly merging with the existing traditional face-to-face teaching-learning methods.

### Declaration of Interest:

E.J., G.G., and V.S. have no interests to declare. G.A. and T.P. were participants of the study. They were not involved in protocol development or data analysis.

## Figures and Tables

**Table 1.** Comparison of Various Social Characteristics Among the 3 Years of Residency

	Year I	Year II	Year III	*P*
Age (mean ± SD)	25.85 ± 1.98	26.91 ± 2.21	28.58 ± 2.81	.005*
% Female	90	54.55	83.33	.066
Relationship (n)				
Single, staying alone	13	9	6	.278
Single, staying with roommate	2	1	4	
Living with boyfriend or girlfriend	2	0	2	
Married, staying alone	0	1	0	
Married, staying with family	1	0	0
Other	2	1	0	
NEET Rank (n)				
<10 000	0	1	1	.230
10 000-15 000	0	1	3	
15 000-20 000	2	3	7	
20 000-30 000	14	6	0	
>30 000	4	0	1
% taken loan	45	63.64	33.33	0.368
Stress (mean ± SD) (n)	2.89 ± 1.17 (9)	3.71 ± 1.11 (7)	1.75 ± 2.06 (4)	.312
% doing 36-48 hours per week	80	100	91.67	.238
% doing > 4 cases	10	9.09	8.33	.091

SD, standard deviation; n, number of residents; NEET, National Eligibility Entrance Test.

*P < .05, significant.

**Table 2.** Cronbach’s Alpha Coefficients for Subscales and Full DREEM Inventory

Variable	n	Cron
Total	50	0.91
SPL	12	0.88
SPT	11	0.7
SAP	8	0.64
SPA	12	0.76
SSP	7	0.71
SAP Minus	7	0.77
FAS	9	0.85
Total Minus	49	0.92

SPL, students’ perception of learning; SPT, students’ perception of teacher; SAP, students’ academic self-perception; SPA, students’ perception of atmosphere; SSP, students’ social self-perception; FAS, faculty assessment score; Cron, Cronbach’s alpha.

**Table 3.** Comparison of Total Scores of DREEM Score and FAS (Mean ± SD [95% CI]) Among the 3 Groups

Variable	Year I	Year II	Year III	*P*
SPL	41.55 ± 5.11 (39.31-43.79)	35.45 ± 6.77 (31.45-39.45)	38.67 ± 4.52 (36.11-41.23)	.092
SPT	36.75 ± 4.89 (34.61-38.89)	34.18 ± 4.33 (31.62-36.74)	34.67 ± 2.64 (33.18-36.16)	.033
SAP	21.5 ± 3.76 (19.85-23.15)	24.18 ± 4.05 (21.79-26.57)	25.42 ± 2.11 (24.23-26.61)	.196
SPA	34.05 ± 5.72 (31.54-36.56)	30.64 ± 7.89 (25.98-35.3)	31.75 ± 4.56 (29.17-34.33)	.016
SSP	20.45 ± 5.45 (18.06-22.84)	17.18 ± 3.49 (15.12-19.24)	19.25 ± 3.6 (17.21-21.29)	.213
C.SAP	21.4 ± 3.72 (19.77-23.03)	20.82 ± 3.66 (18.66-22.98)	22.25 ± 1.76 (21.25-23.25)	.106
Total	154.3 ± 20.8 (145.18-163.42)	141.64 ± 22.41 (128.4-154.88)	149.75 ± 9.49 (144.38-155.12)	.484
C.Total	154.2 ± 20.73 (145.11-163.29)	138.27 ± 22.12 (125.2-151.34)	146.58 ± 9.28 (141.33-151.83)	.042*
FAS	32.55 ± 3.39 (31.06-34.04)	29.82 ± 4.47 (27.18-32.46)	31.42 ± 2.5 (30.01-32.83)	.135

DREEM, Dundee Ready Education Environment Measure; SPL, students’ perception of learning; SPT, students’ perception of teacher; SAP, students’ academic self-perception; SPA, students’ perception of atmosphere; SSP, students’ social self-perception; C.SAP, corrected SAP; C.Total, corrected total; FAS, faculty assessment score; SD, standard deviation.

*P* < .01, significant; * *P* < .05, significant for total and C.Total (CI 95% for total and C.Total).

**Table 4.** Comparison of Scores of Individual Domains Among the 3 Groups (*P*)

	Y1 vs Y2	Y1 vs Y3	Y2 vs Y3
Total*	.057	.520	.056
SPL	.016	.138	.164
SPT	.120	.204	.420
SAP	.115	.005	.305
SPA	.126	.242	.370
SSP	.054	.309	.153
C.SAP	.632	.518	.173
FAS*	.064	.231	.351
C.Total*	.027*	.259	.052

SPL, students’ perception of learning; SPT, students’ perception of teacher; SAP, students’ academic self-perception; SPA, students’ perception of atmosphere; SSP, students’ social self-perception; C.SAP, corrected SAP; C.Total, corrected total; FAS, faculty assessment score.

*P < .05 is considered significant. For other values *P* < .01 is considered significant. *P* < .01, significant.

**Table 5.** Average Scores (Score/Number of Questions) (Mean ± SD)

VAR	Y1	Y2	Y3
Total	3.13 ± 0.52	2.83 ± 0.5	2.99 ± 0.51
SPL	3.46 ± 0.32	2.95 ± 0.42	3.22 ± 0.37
SPT	3.34 ± 0.51	3.11 ± 0.54	3.15 ± 0.55
SAP	2.95 ± 0.49	3.02 ± 0.45	3.39 ± 0.42
SPA	2.84 ± 0.58	2.55±0.35	3.18 ± 0.38
SSP	2.92 ± 0.38	2.45 ± 0.5	2.65 ± 0.5
C.SAP	2.85 ± 0.49	2.71 ± 0.5	2.91 ± 0.38
FAS	3.62 ± 0.24	3.31 ± 0.37	3.49 ± 0.23
C.TOTAL	3.03 ± 0.52	2.52 ± 0.5	2.84 ± 0.51
*P *(Kendall’s *W*)*	.000 (0.37)**	.002 (0.38)**	.000 (0.46)**

SPL, students’ perception of learning; SPT, students’ perception of teacher; SAP, students’ academic self-perception; SPA, students’ perception of atmosphere; SSP, students’ social self-perception; C.SAP, corrected SAP; C.Total, corrected total; FAS, faculty assessment score; SD, standard deviation.

*Difference among domains of DREEM score. ** *P* < .01, significant.
